# Community-led delivery of HIV self-testing to improve HIV testing, ART initiation and broader social outcomes in rural Malawi: study protocol for a cluster-randomised trial

**DOI:** 10.1186/s12879-019-4430-4

**Published:** 2019-09-18

**Authors:** Pitchaya P. Indravudh, Katherine Fielding, Moses K. Kumwenda, Rebecca Nzawa, Richard Chilongosi, Nicola Desmond, Rose Nyirenda, Cheryl C. Johnson, Rachel C. Baggaley, Karin Hatzold, Fern Terris-Prestholt, Elizabeth L. Corbett

**Affiliations:** 1 Department of Global Health and Development, Faculty of Public Health and Policy, London School of Hygiene & Tropical Medicine, London, UK; 2 Malawi-Liverpool-Wellcome Trust Clinical Research Programme, Blantyre, Malawi; 3 Department of Infectious Disease Epidemiology, London School of Hygiene & Tropical Medicine, London, UK; 4 Population Services International, Lilongwe, Malawi; 5 Clinical Sciences Department, Liverpool School of Tropical Medicine, Liverpool, UK; 6 Department of HIV and AIDS, Ministry of Health, Lilongwe, Malawi; 7 Department of HIV/AIDS, World Health Organisation, Geneva, Switzerland; 8 Population Services International, Johannesburg, South Africa; 9 Department of Clinical Research, London School of Hygiene & Tropical Medicine, London, UK

**Keywords:** HIV, HIV testing, HIV self-testing, Community-led, Community mobilisation, Adolescents, Men, Malawi

## Abstract

**Background:**

Prevention of new HIV infections is a critical public health issue. The highest HIV testing gaps are in men, adolescents 15–19 years old, and adults 40 years and older. Community-based HIV testing services (HTS) can contribute to increased testing coverage and early HIV diagnosis, with HIV self-testing (HIVST) strategies showing promise. Community-based strategies, however, are resource intensive, costly and not widely implemented. A community-led approach to health interventions involves supporting communities to plan and implement solutions to improve their health. This trial aims to determine if community-led delivery of HIVST can improve HIV testing uptake, ART initiation, and broader social outcomes in rural Malawi.

**Methods:**

The trial uses a parallel arm, cluster-randomised design with group village heads (GVH) and their defined catchment areas randomised (1:1) to community-led HIVST or continue with the standard of the care (SOC). As part of the intervention, informal community health cadres are supported to plan and implement a seven-day HIVST campaign linked to HIV treatment and prevention. Approximately 12 months after the initial campaign, intervention GVHs are randomised to lead a repeat HIVST campaign. The primary outcome includes the proportion of adolescents 15–19 years old who have tested for HIV in their lifetime. Secondary outcomes include recent testing in adults 40 years and older and men; ART initiation; knowledge of HIV prevention; and HIV testing stigma. Outcomes will be measured through cross-sectional surveys and clinic registers. Economic evaluation will determine the cost per person tested, cost per person diagnosed, and incremental cost effectiveness ratio.

**Discussion:**

To the best of our knowledge, this is the first trial to assess the effectiveness of community-led HTS, which has only recently been enabled by the introduction of HIVST. Community-led delivery of HIVST is a promising new strategy for providing periodic HIV testing to support HIV prevention in rural communities. Further, introduction of HIVST through a community-led framework seems particularly apt, with control over healthcare concurrently devolved to individuals and communities.

**Trial registration:**

Clinicaltrials.gov registry (NCT03541382) registered 30 May 2018.

**Electronic supplementary material:**

The online version of this article (10.1186/s12879-019-4430-4) contains supplementary material, which is available to authorized users.

## Background

### HIV epidemic

Prevention of new HIV infections is a critical public health issue. In 2017, 1.6 million adults were newly infected with HIV, with two-thirds in sub-Saharan Africa [1]. Global strategies to reduce HIV incidence aim to maximise early diagnosis, treatment and viral suppression of people living with HIV [2]. Regional expansion of facility-based HIV testing services (HTS) has contributed to declining HIV incidence, but one-fifth of people with HIV aged 15–64 years old remain undiagnosed [1]. The highest HIV testing gaps are in men, adolescents 15–19 years old, and adults 40 years and older, contributing to ongoing HIV transmission and poorer outcomes from late HIV diagnosis [3, 4]. Barriers to uptake of facility-based HTS include stigmatising norms, discrimination from health care workers, distance to health facilities, and direct and indirect costs of service utilisation [5].

### Community-based HIV testing and self-testing

Community-based HTS can contribute to increased testing coverage, early HIV diagnosis, and reduced HIV incidence [6–9], with HIV self-testing (HIVST) strategies showing promise [10–12]. In 2016, HIVST was recommended by WHO as an additional approach to providing HTS based on evidence of high acceptability, feasibility, accuracy and uptake [13]. In urban Malawi, distribution of HIVST kits by community volunteers achieved high uptake and accuracy, with increased demand for antiretroviral therapy (ART) with offer of home-based care [10, 14]. Home-based HIVST in rural Malawi increased recent testing by 20%, including in men and adolescents, beyond the percentage achieved by facility-based HTS [11]. The addition of HIVST kit distribution to home-based HTS provided by community health workers (CHW) in urban Zambia further increased knowledge of status by 3%, with a difference in intervention effect by sex [12]. Low adverse events were reported across studies [12, 15].

Community-based strategies, however, are resource intensive, costly and not widely implemented. In population-based surveys, a low percentage of respondents indicate most recently testing through community-based services [16]. Societal costs of community-based HTS and HIVST tend to be lower than facility-based HTS, but providers costs are consistently higher, especially the cost per new HIV diagnosis [7, 17–19].

### Community-led approaches to improve health

A community-led approach (also known as community mobilisation, community-directed or community-driven approaches) to health interventions involves supporting communities to develop the knowledge and skills to identify problems contributing to poor health, plan and implement solutions to improve their health, and evaluate implementation of solutions [20, 21].

Most practice uses participatory learning and action methods, which involve engaging groups of individuals to identify their needs, understand the root causes of these needs, and translate awareness into action [22]. From an organisation and service delivery perspective, community participation in the design and management of health programmes can enhance their coverage, efficiency and equity through context-driven decision-making and resource allocation [23]. The change process is based on a number of assumptions, namely that individuals desire to be involved in decisions about their own healthcare and will contribute resources to improve community health; individuals will be more likely to change their attitudes and behaviour as a result of their involvement; and individuals will be empowered through knowledge, skills and confidence gained through their involvement [24, 25].

Evaluations of community-led programmes across multiple disease areas report evidence of improved health outcomes at similar or lower cost to vertical programmes, with applications in onchocerciasis [26], dengue [27–29], HIV [30, 31], maternal and child health [32–40], and sanitation [41, 42]. Within HIV, community-led programmes have involved outreach events to promote HIV prevention or provision of HTS within multi-disease campaigns [30, 31, 43]. Most studies also evaluate delivery of vertically-defined interventions and disease areas through community-driven systems, with community motivation to deliver externally-prescribed interventions often contingent on the severity of the perceived risk of disease and value of the intervention to the health and well-being of the community [44].

### Rationale for randomised trial

The types of interventions that can be delivered by communities is expanding with increasing availability of novel self-care technologies. This trial aims to determine whether community-led delivery of HIVST can increase uptake of HIV testing, ART initiation, and broader social outcomes in a high HIV-burden setting in rural Malawi. While prior randomised trials have established the impact of vertically-delivered, community-based HIVST models on uptake of HIV testing, it is uncertain whether similar outcomes could be achieved when increasing responsibility for the design and management of HIVST implementation is devolved to communities. In addition to distribution of kits, HIVST implementation involves consideration around linkage to routine HIV services and potential social harm that can be difficult to support in resource-poor contexts and warrant further evaluation under a randomised trial.

## Methods/design

### Aim

The primary aim of this trial is to test whether community-led delivery of HIVST in rural Malawi can increase the proportion of the population who has tested for HIV compared to the standard of care (SOC), with a focus on underserved sub-groups including adolescents 15–19 years old, older adults 40 years and older, and men. The trial also aims to assess the impact of community-led HIVST on ART initiation and broader social outcomes.

### Study design

**Fig. 1:**
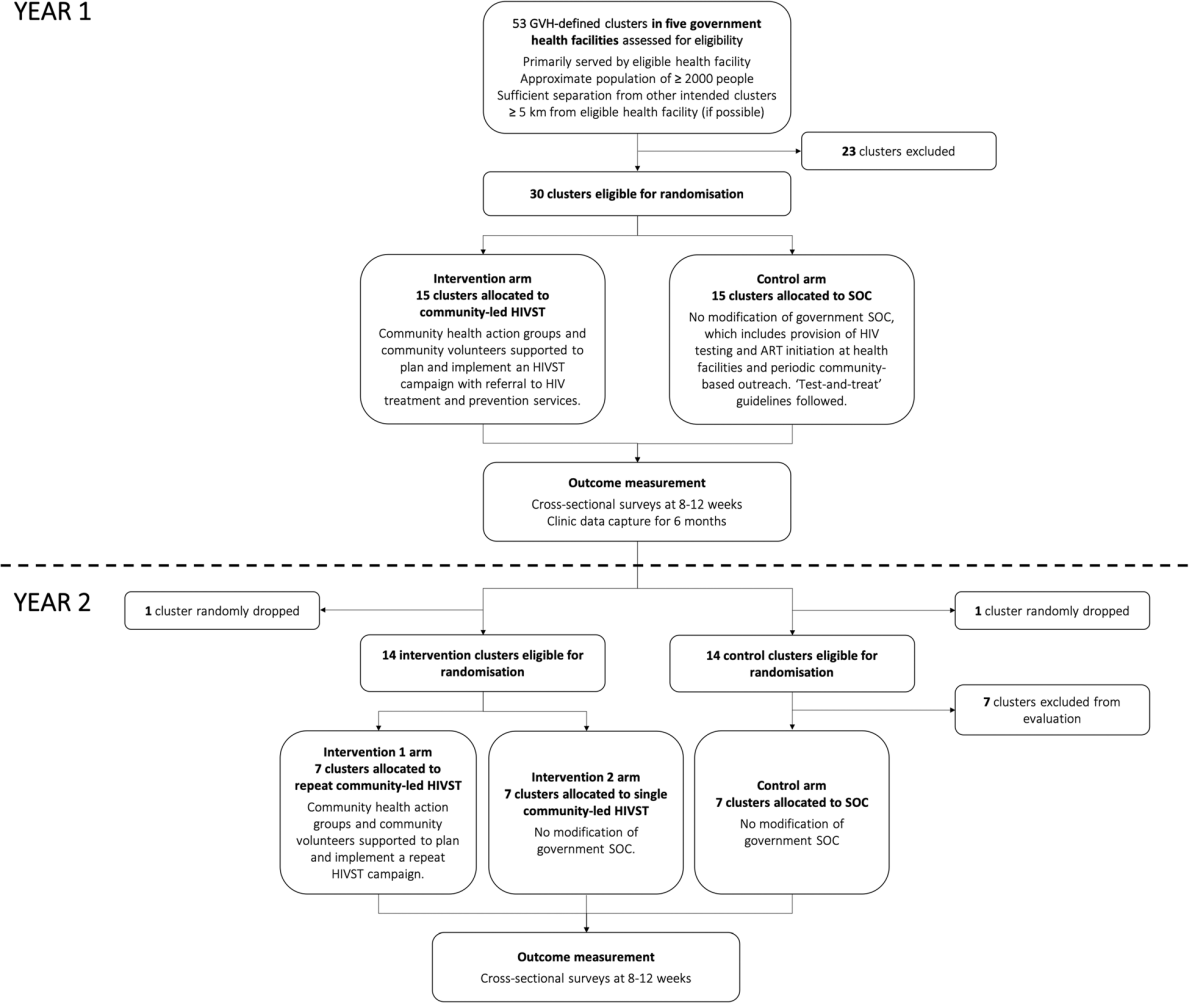
Flow diagram of trial design

The trial uses a parallel arm, cluster-randomised design with two stages of randomisation (Fig. 1). Clusters are defined as group village heads (GVH) and their respective catchment areas, thereafter referred to as GVHs. The first stage includes two arms, with 30 GVHs randomised (1:1) to community-led HIVST or continue with the SOC. As part of the intervention, community health action groups and community volunteers plan and implement an HIVST campaign linked to HIV treatment and prevention services in their areas.

The second stage comprises of a three-arm study with 21 GVHs. Fourteen of 15 GVHs receiving the community-led HIVST arm in the first stage are randomised (1:1) to lead a repeat HIVST campaign approximately 12 months after the initial campaign or not deliver a repeat campaign. Fourteen of 15 GVHs from the SOC arm are randomised to be included in or excluded from the second stage of the study.

The trial began in October 2018 and will be completed in December 2019.

### Study setting and population

The trial takes place in the catchment areas of five government health facilities in Mangochi district, which has among the highest poverty rates and lowest educational attainment in the country. In 2016, Mangochi had HIV prevalence of 13.2% in women and 5.7% in men [45]. Coverage of lifetime testing and testing in the last 12 months was, respectively, 70.9 and 36.2% in women and 58.2 and 38.1% in men [45].

Most areas in Malawi are organized by traditional chieftaincy systems. GVHs have customary authority over a group of villages, while community health action groups serve as representatives of community health issues at GVH-level [46]. Community health action groups oversee provision of services by village-level cadres, including village health committees and community health volunteers. CHWs attached to government health facilities liaise with community health action groups on delivery of community health services. In practice, the organisational and operational capacity of community health structures vary widely.

**Fig. 2:**
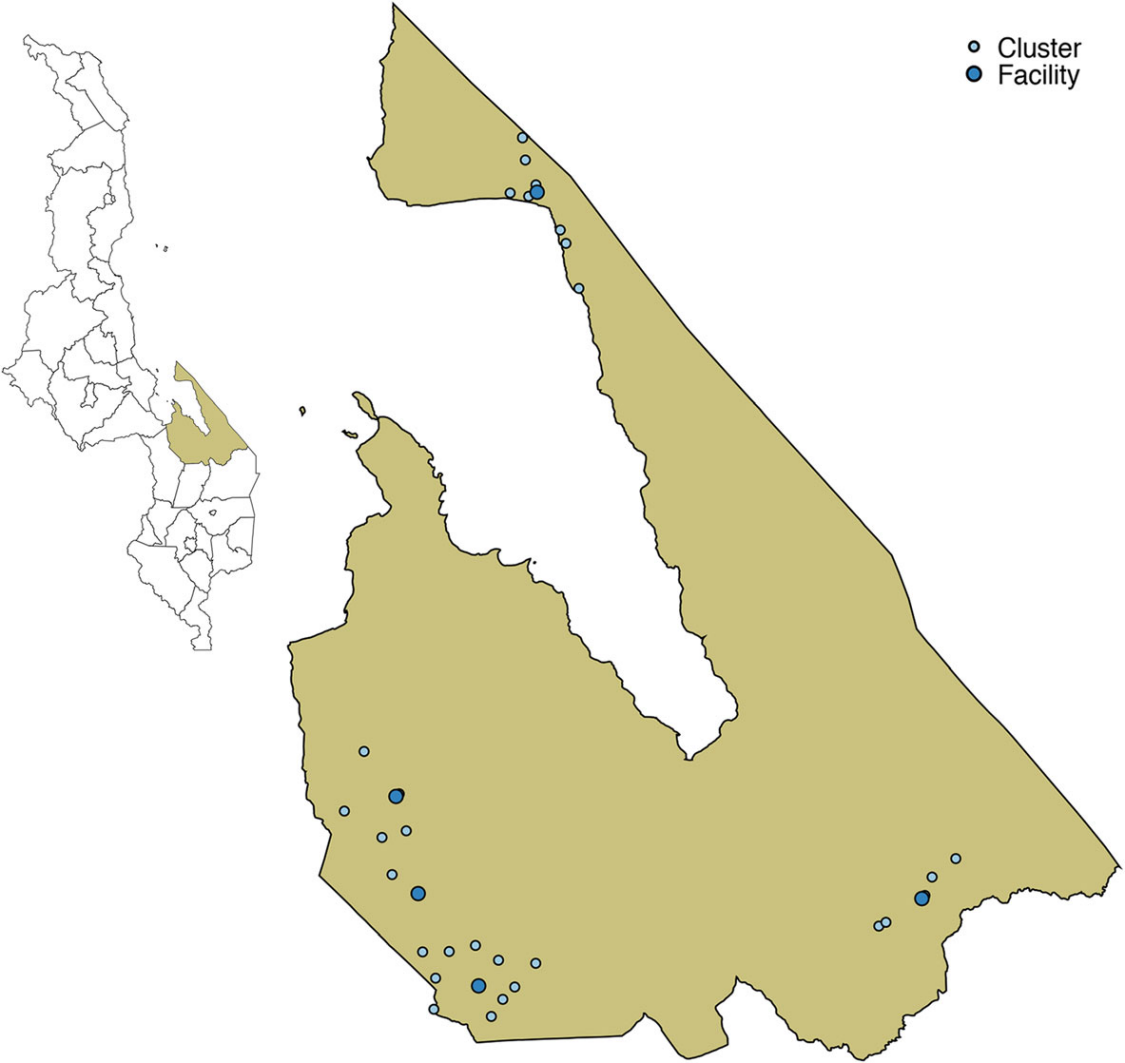
Trial clusters in Mangochi district. Map of Mangochi district, Malawi with trial health facilities and group village head-defined clusters. Data source: data.humdata.org and primary data

GVHs were considered eligible for the study if they were: (i) primarily served by an eligible government health facility for HIV testing and ART services, (ii) responsible for an approximate catchment population of at least 2000 people, (iii) sufficiently separated from boundaries of other intended clusters, and (iv) at least five kilometres away from an eligible health facility, if possible. All adults aged 15 years and older within GVHs were eligible for the evaluation. Figure 2 includes a map of Mangochi district, with health facilities and GVHs included in the trial.

### Study procedures

#### SOC

The SOC is defined based on HIV services currently provided by the Ministry of Health. In Malawi, HIV testing and ART services are provided at most health facilities and through periodic community-based outreach. HIV testing is administered using finger-prick rapid diagnostic tests based on the national testing algorithm. Universal “test-and-treat” guidelines are followed.

#### Community-led HIVST

**Fig. 3:**
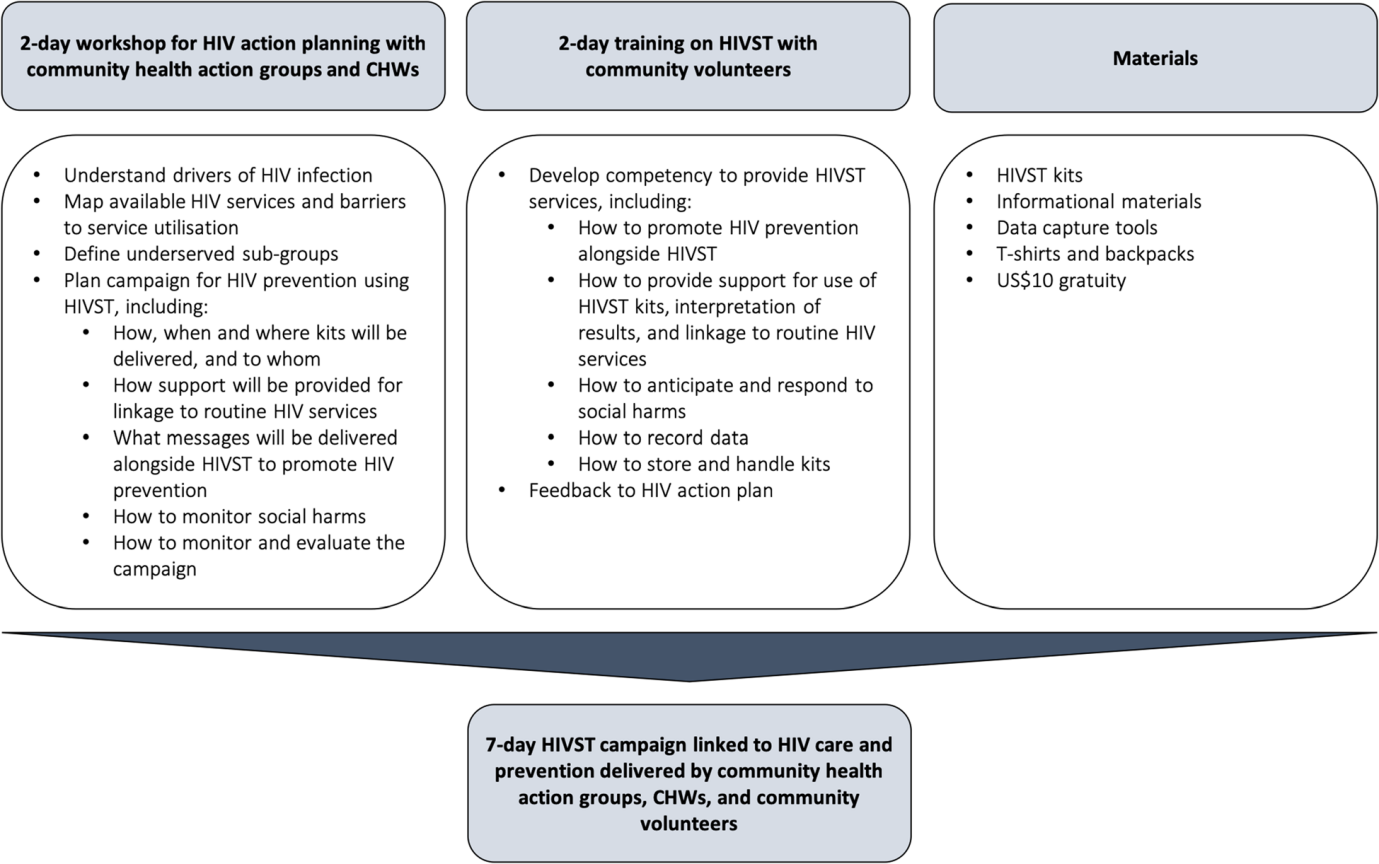
Intervention design

The community-led HIVST intervention consists of (i) participatory workshops for action planning with community health action groups and CHWs, (ii) trainings on HIVST promotion and support with village-level community volunteers, and (iii) HIVST campaigns linked to HIV treatment and prevention (Fig. 3). The framework for the intervention design is modelled after previous community mobilisation interventions, which utilise participatory learning and action methods for critical reflection and action [22]. The final design was informed by focus group discussions with community residents, workshops with representatives from the Department of HIV/AIDS, and piloting prior to the trial (Additional file 1). The intervention is overseen by the implementation team, which include the Malawi-Liverpool-Wellcome Trust Clinical Research Programme, Population Services International (PSI) Malawi and the Ministry of Health.

Community health action groups and CHWs attend two-day participatory workshops. The aim of the workshops is to mobilise existing community health structures and support them in planning and delivering HIVST campaigns in their catchment areas. As part of the workshops, community health action groups and CHWs identify drivers of HIV infection, map available HIV services and barriers to service utilisation, define sub-groups underserved by HIV services, and develop a context-driven campaign. Specifically, they are tasked with deciding how, when and where HIVST kits will be delivered and to whom; how self-testers will be supported to link to routine HIV care and prevention services; what messages will be delivered alongside HIVST to promote HIV prevention; how to monitor social harms related to HIVST; and how to monitor and evaluate the campaign.

Community volunteers also attend two-day trainings on HIVST promotion and support. Volunteers are trained in how to provide information and support for use of HIVST kits, interpretation of results, and linkage to routine services (confirmatory testing and ART initiation for reactive results, voluntary medical male circumcision [VMMC] for men with non-reactive results, couples testing for serodiscordant partners). Volunteers also receive training in how to provide information around HIV prevention, including effectiveness of ART and VMMC and prevention within serodiscordant partners. Lastly, volunteers are trained in how to anticipate and respond to social harms, record data, and handle and store kits.

Community volunteers then implement seven-day HIVST campaigns linked to HIV treatment and prevention, with supervision by community health action groups and CHWs. The campaign period is based on the typical length for HTS campaigns under the Ministry of Health. The project team provide HIVST kits (OraQuick HIV Self-Test), informational materials, and data capture tools. Community health action groups and volunteers receive US$10 gratuity per campaign as nationally standardised for informal community health cadres. Adults aged 15 years and older are eligible for HIVST and can take multiple kits if desired.

#### Repeat community-led HIVST

Approximately 12 months after the initial HIVST campaign, GVHs allocated to the repeat community-led HIVST arm will plan and implement an additional campaign, with a similar intervention package provided by the implementation team.

### Outcomes

For the first-stage evaluation, the primary outcome includes:

- Proportion of adolescents 15–19 years old who have tested for HIV in their lifetime.

Secondary outcomes include:

- Proportion of older adults 40 years and older who have tested in the last 3 months.
- Proportion of men who have tested in the last 3 months.
- Cumulative incidence of ART initiation across 6 months.
- Measure of knowledge of HIV prevention methods.
- Measure of perceived HIV testing stigma.

Outcomes will be measured through cross-sectional surveys administered 8–12 weeks after the start of the community-led HIVST intervention, with matched calendar time in both study arms. ART initiation will be captured by clinic assistants stationed at the nearest health facility for 6 months following the intervention start date.

For the second-stage evaluation, the primary outcome includes:

- Proportion of individuals who have tested for HIV in the last 12 months.

Secondary outcomes include:

- Proportion of individuals who have initiated on ART in the last 12 months.
- Measure of knowledge of HIV prevention methods.
- Measure of perceived HIV testing stigma.

Outcomes will be measured through surveys administered 8–12 weeks after the start of the repeat community-led HIVST intervention.

### Sample size

To calculate the sample size for the first-stage evaluation, we assumed that the proportion of lifetime testing for adolescents aged 15–19 years old in the SOC arm was 35–50% based on the 2015–16 Malawi Demographic and Health Survey [45]. With 15 clusters per arm and 50 adolescents per cluster, we will have at least 90% power to detect a 20% absolute increase in lifetime testing using a coefficient of variation of outcomes (k) of 0.25. With adolescents making up 20% of the adult population, this will require 250 respondents per cluster.

For the second-stage evaluation, we assumed testing in the last 12 months ranged between 30 and 40%. With seven clusters per arm and 250 respondents per cluster, we have at least 90% power to detect a 25% absolute difference in recent testing between the repeat community-led HIVST and SOC arms, assuming k = 0.25.

### Randomisation and blinding

For the main trial, 30 GVHs were randomised 1:1 to community-led HIVST or SOC. The second stage involves 1:1 randomisation of 14 of the 15 clusters in the community-led HIVST arm to deliver a repeat or single HIVST campaign, and 1:1 randomisation of 14 of the 15 clusters in the SOC arm to be included or excluded from the second-stage evaluation. GVHs were assigned to study arms at a public ceremony. Three balls numbered 0–9 were selected from an opaque bag, corresponding to one of 1000 randomisation combinations. Restricted randomisation was used to ensure balance between arms based on the nearest health facility, distance from the health facility, population and number of villages. Study staff are blinded from the study allocation status as much as possible, with all data managed without reference to arms.

### Data collection

#### Outcome evaluation

##### Cross-sectional survey

**Fig. 4:**
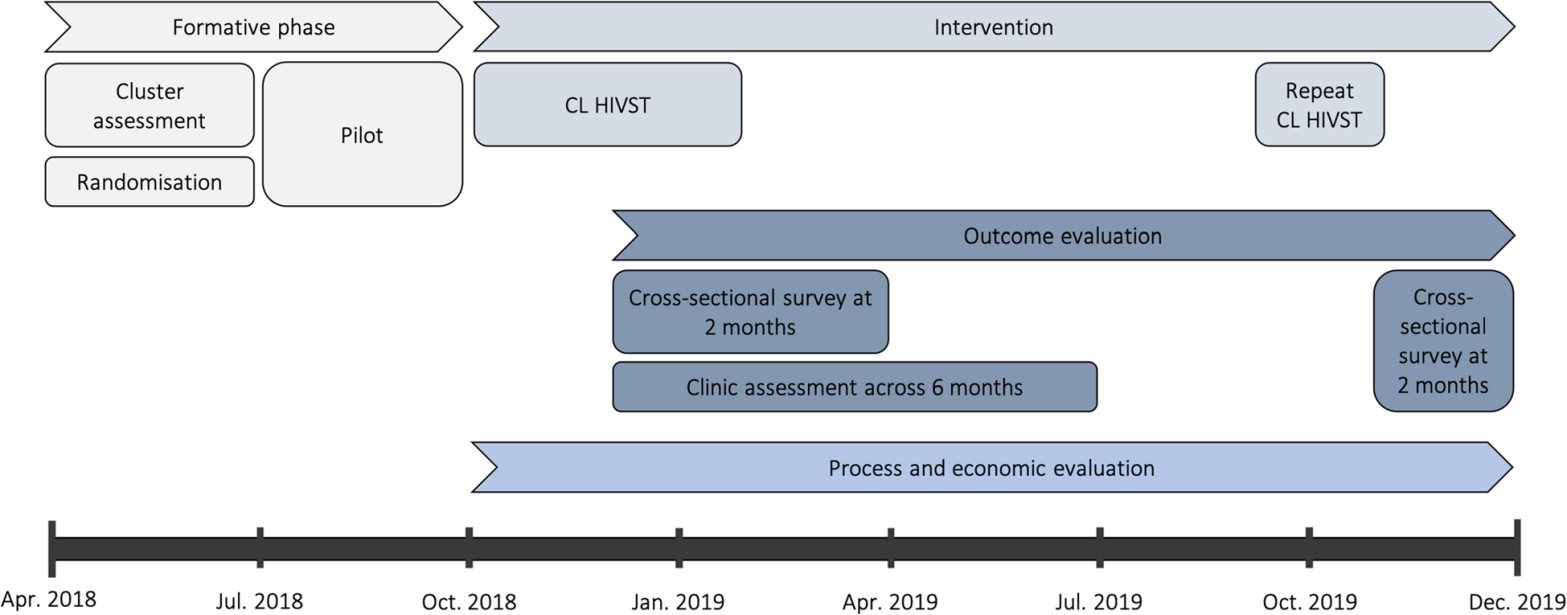
Trial timeline

Cross-sectional surveys will be administered in two rounds approximately 8–10 weeks after the respective starts of the initial and repeat community-led HIVST interventions (Fig. 4). For each GVH, separate evaluation villages for the first and second survey will be randomly selected from villages with at least a population of 500 people and located centrally within the catchment area. All households in the evaluation villages will be eligible to participate in the survey and enumerated, with the exception of villages with more than 250 adults, where 150 households will be enumerated starting with the village head household and proceeding in a clockwise spiral. Inclusion criteria for the survey include aged 15 years and older and resident in eligible households. Written consent will be obtained for all participants, except participants aged 15–17 years old, who will be asked to assent and their parent or guardian asked to consent (Additional file 2).

All respondents will complete a brief individual questionnaire with modules on sociodemographic characteristics; prior HIV testing, self-testing, treatment and prevention; and sexual behaviour. The head of household or representative will also complete a module on household characteristics. A random sample of respondents (approximately 20%) will receive an extended questionnaire on community mobilisation, knowledge of HIV prevention methods, and HIV testing stigma.

##### Clinic registers

ART initiation will be captured for the 6-month period following the start of the first stage of the trial. Clinic assistants at the five study health facilities will establish eligibility of all incoming ART patients, which include aged 15 years and older, resident in study clusters, and starting or re-starting on ART. Sociodemographic characteristics, prior HIVST, and ART status of eligible patients will be recorded on study forms.

#### Process evaluation

Quantitative and qualitative data will be collected to understand implementation and context related to the community-led HIVST intervention [47]. To investigate how the intervention was implemented and adaptations by cluster, data will be collected on the sociodemographic characteristics of community health action groups and community volunteers; attendance by community health action groups and volunteers in workshops, trainings and HIVST campaigns; and activities planned and implemented during the campaign. Reach will be assessed using HIVST registers, which track sociodemographic background and prior HIV testing of residents collecting HIVST kits. Reports of linkage to routine HIV services as well as social harms will also be recorded. Finally, participant observations and semi-structured interviews will be conducted to explore how communities perceived and interacted with the intervention and how the intervention and implementation were influenced by contextual factors.

#### Economic evaluation

Financial and economic data will be collected to determine the cost per person tested and diagnosed under the community-led HIVST intervention and SOC, and the incremental cost-effectiveness ratio of the intervention. Methods are drawn from global guidelines on costing of health interventions [48]. A societal perspective will be used to capture costs to providers, communities and individuals. A combination of ‘top-down’ and ‘bottom-up’ costing approaches will be used, with financial costs from analysis of expenditures supplemented with full costs obtained through direct observations, individual interviews, and review of databases and records. Number of tests and HIV-positive diagnoses will be obtained through extraction of HTS and HIVST registers.

### Data management

Quantitative data will be captured using electronic tablets and optical character recognition forms routinely entered into a dedicated database. Data will be queried regularly for errors or inconsistencies and followed-up according to quality assurance standard operating procedures. Missing data will also be examined by variable and observation to ascertain the quantity of missing data and patterns of missingness. Qualitative data will be recorded using observational notes and digital audio recordings, which will be transcribed and translated into English. Study respondents providing written consent will be assigned an identification number, with names linked through paper-based recruitment logs stored in locked filing cabinets.

### Statistical analysis

Data analysis for primary and secondary outcomes will be based on an intention-to-treat using methods appropriate for cluster-randomised designs [49]. Covariates, including but not restricted to sex, age, marital status, educational attainment and wealth status, will be summarised by study arm to assess for any imbalance. Trial outcomes will be analysed at a cluster-level, giving each cluster equal weight. The overall outcome risk for each cluster will be calculated, and a log transformation will be applied to the summary value for each cluster if necessary. The mean and standard deviation of these log risks will be used to obtain the geometric mean and associated 95% confidence interval for each study arm. The risk difference will be calculated for the primary outcome. The risk ratio, 95% confidence interval and *p*-value will also be estimated from t-tests.

Adjusted analysis will use a two-stage approach. Logistic regression will be used to adjust for confounding bias at the individual level, and calculate expected events. The ratio of observed to expected events will then be calculated for each cluster, and log-transformed if appropriate. A t-test of the log ratio by arm will be used to estimate the adjusted risk ratio, 95% confidence interval and *p*-value.

A systematic assessment of missingness will be conducted. Sensitivity analysis will compare complete case analysis results with those where missing data are imputed. A full statistical analysis plan will be developed prior to unblinding of data.

### Results dissemination

The results of this trial will be distributed to global and national policy makers. Ministry of Health representatives are collaborators on this trial and have advised on the scope of research to ensure its relevance to national policy development. Feedback sessions will also be held with community representatives from participating trial sites.

### Social harms

Social harms will be captured by community health action groups and community volunteers using programme registers. Reported social harms will be monitored, categorised based on an established grading system [50], followed-up by the project team, and reported to the trial governance and ethics review committees if appropriate. Social harms will also be assessed through cross-sectional surveys.

### Trial governance, ethics approvals and funding

Oversight of the trial is conducted by an independent technical advisory group (TAG), which consists of six public health experts, scientists and policy makers guiding research under STAR. The TAG meets semi-annually to review progress, data and adverse events from ongoing studies. A separate data and safety monitoring board was not established given that HIVST is well-established and low risk [13]. The trial is subject to audits from the London School of Hygiene & Tropical Medicine (LSHTM) under their remit as sponsor.

The trial protocol has been approved by research ethics committees at the University of Malawi College of Medicine (ref: P.01/18/2332), LSHTM (ref: 14761), and WHO (ref: STAR-comm led CRT-Malawi), with the latter submission process involving peer review. The trial is registered with ClinicalTrials.gov (ref: NCT03541382). Funding is primarily supported by Unitaid, who is independent of the design, management, analysis and reporting of the trial.

## Discussion

This trial aims to determine if community-led delivery of HIVST can improve HIV testing uptake, ART initiation, and broader social outcomes in rural Malawi. The intervention also aims to address current implementation gaps related to coverage of HIV testing in adolescents 15–19 years, older adults 40 years and older, and men; resources required for delivering community-based services; and community engagement in HIV prevention. To the best of our knowledge, this is the first trial to assess the effectiveness of community-led HTS, which has only recently been enabled by the introduction of HIVST. The trial builds on earlier studies evaluating ‘top-down’ community-based HTS and HIVST [6–12] and ‘bottom-up’ community-led outreach for HIV prevention [30], which have shown increased uptake of HIV testing, early detection of people living with HIV, and reduction in HIV incidence.

The intervention evaluated in this trial consists of three components implemented across a two-week period: (i) participatory workshops for action planning, (ii) trainings on HIVST promotion and support, and (iii) HIVST campaigns linked to HIV treatment and prevention. Previous evaluations of community-led programmes have described the importance of the participatory process [51, 52], which aims to facilitate dialogue and connection among groups of individuals towards a collective sense of awareness, solidarity and agency, and enable groups to take action to address factors contributing to poor health [53]. We hypothesise that introduction of HIVST within a community-led framework could improve access to HIV services and health education on HIV testing, treatment and prevention. Potential gains from repeat campaigns will also be evaluated. Periodicity is an important consideration, with more frequent implementation potentially lowering costs but delivering diminishing returns. Further, long-term community engagement could contribute to improved community capacity to address their own health problems as well as influence broader social norms, including around HIV prevention.

Our intervention aims to facilitate community action around HIV prevention using HIVST. As part of the intervention, communities are supported to develop strategies to promote messaging around HIV prevention and linkage to ART initiation for reactive results, VMMC for men with non-reactive results, and couples testing for serodiscordant partners. Linking HIVST with HIV prevention strategies is critical for maximising the health impact of HIV testing, especially with declining HIV positivity. At the same time, HIVST could be used to generate demand for HIV prevention at individual and community levels [54].

This trial will provide evidence on an alternative model for community-based HTS that could be adopted in settings with established community health structures. Underlying this trial is the question of whether informal community health cadres in rural sub-Saharan Africa can effectively lead the design and management of HIVST implementation. Provision of HIVST involves multiple components, including distribution of kits, education on correct use of kits, support for linkage to routine HIV services, safety monitoring, and data capture and assessment. At best, shifting responsibility for HIVST implementation to communities could result in the aforementioned health and social benefits and cost reductions. At worst, misdiagnosis, loss to follow-up and social harm could arise from poor-quality implementation, compromising gains in health. The burden of implementation could place further economic costs on resource-constrained communities [26, 55]. Elite capture, whereby socially and economically privileged sub-groups are favoured in resource allocation, could also perpetuate existing health disparities [56].

This trial has a number of anticipated limitations. First, the SOC arm is defined by the standard HTS package provided by the Ministry of Health, which includes facility-based HTS and recurring community-based outreach, rather than parallel community-based HTS or HIVST campaigns. As a result, the effect of the community-led and HIVST intervention components may be difficult to isolate. Second, trial outcomes cannot be adjusted for cluster-level differences between arms at baseline since data were not collected prior to implementation. Third, we anticipate wide cluster-level adaptation of implementation, with our process data critical to understanding any outcome variation.

In summary, the primary aim of this trial is to test whether community-led delivery of HIVST in rural Malawi can increase the proportion of the population who has tested for HIV compared to the SOC, with a focus on underserved sub-groups. The trial also aims to assess the impact of community-led HIVST on ART initiation and broader social outcomes. Community-led HIVST is a promising new strategy for providing periodic HIV testing to support HIV prevention in rural communities. Further, introduction of HIVST through a community-led framework seems particularly apt, with control over healthcare concurrently devolved to individuals and communities.

## Additional files

Additional file 1: Description of intervention design. Summary of findings from the formative research and pilot to inform the intervention design. (DOCX 16 kb)

Additional file 2: Participant information sheet, consent form and assent form. Participant information sheet, consent form and assent form for the cross-sectional survey. (DOCX 31 kb)

## Data Availability

Trial data will be stored on LSHTM and MLW servers. Anonymised data will also be shared through a public data repository or directly with other researchers upon request. PPI should be contacted for data requests.

## Abbreviations

AIDS: Acquired immunodeficiency syndrome
ART: Antiretroviral therapy
CHW: Community health worker
GVH: Group village head
HIV: Human immunodeficiency virus
HIVST: HIV self-testing
HTS: HIV testing services
LSHTM: London School of Hygiene & Tropical Medicine
PSI: Population Services International
SOC: Standard of care
STAR: HIV Self-Testing Africa
TAG: Technical advisory group
VMMC: Voluntary medical male circumcision
WHO: World Health Organisation
