# Supplementary material for: Community-led delivery of HIV self-testing to improve HIV testing, ART initiation and broader social outcomes in rural Malawi: study protocol for a cluster-randomised trial
Source: BMC Infect Dis. 2019 Sep 18;19:814. doi: 10.1186/s12879-019-4430-4 (PMC6751650; doi:10.1186/s12879-019-4430-4)
Supplement: Supplementary file 1 — Description of intervention design. Summary of findings from the formative research and pilot to inform the intervention design. (DOCX 16 kb) [file 12879_2019_4430_MOESM1_ESM.docx]

**Additional file 1. Description of intervention design**

|  | **Intervention assessed** | **Results** |
| --- | --- | --- |
| Focus group discussion (n=16) | Not applicable | - Interest demonstrated in leading HIVST campaign. - Close relationship between community members suggested as advantage. Geographic spread and large population size suggested as disadvantages. - Factors seen as critical to implementation included:   - Early community sensitisation   - Committee to oversee implementation, either by community health action group or formed by chief or community   - Engagement of informal community health cadres and CHWs   - Financial compensation |
| Pilot 1 | - Community forum to select HIVST committee - 2-day participatory workshops with HIVST committee to plan HIVST campaign and select community volunteers - 2-day training with community volunteers - 7-day HIVST campaign - No stipend | - 1244 kits distributed (N=2,372, 52.3%); 36.9% to men and 21% to adolescents. - Members outside of community health action group selected to HIVST committee, resulting in creation of a parallel committee. Members also had poor health literacy. - Concepts and activities introduced in workshops and trainings too complex. - Poor supervision of HIVST campaign by committee. - Backlash from HIVST committee and community volunteers for lack of financial compensation. - Low engagement byCHWs. |
| Pilot 2 | - Inclusion of CHWs in entrance meetings and participatory workshops - Simplified 2-day participatory workshop with community health action group to plan HIVST campaign - Community forum to select community volunteers - Simplified 2-day training with community volunteers - 7-day HIVST campaign - Stipend for community health action groups and community volunteers | - 3487 kits distributed (N=6,855, 50.1%); 46.5% to men and 27.7% to adolescents. - CHAGs more literate in HIV-related issues. Resulted in improved planning and implementation of HIVST campaign. - Financial compensation aligned with expectations from prior MoH campaigns. - Community forums proved to be a missed opportunity for distribution of HIVST kits. |
| Final intervention | - Inclusion of CHWs in entrance meetings and participatory workshops - Simplified 2-day workshop with community health action groups to plan HIVST campaign - Simplified 2-day training with village health committees and community health volunteers - 7-day HIVST campaign - Stipend for community health action group and community volunteers |  |

CHW, community health workers; HIVST, HIV self-testing
