# Supplementary material for: Community-led delivery of HIV self-testing to improve HIV testing, ART initiation and broader social outcomes in rural Malawi: study protocol for a cluster-randomised trial
Source: BMC Infect Dis. 2019 Sep 18;19:814. doi: 10.1186/s12879-019-4430-4 (PMC6751650; doi:10.1186/s12879-019-4430-4)
Supplement: Supplementary file 2 — Participant information sheet, consent form and assent form. Participant information sheet, consent form and assent form for the cross-sectional survey. (DOCX 31 kb) [file 12879_2019_4430_MOESM2_ESM.docx]

## Additional file 2. Participant information sheet, consent form and assent form

## Participant information sheet

### 1. Why are we doing this study?

Regular HIV testing is very important in Malawi and worldwide because it helps people with HIV get treatment and it may also help to cut down the spread of HIV. We are interested in making it easy for people to get tested for HIV, and then get treatment if they are HIV-positive or better protection if they are HIV-negative. HIV self-testing is a way for people to test themselves for HIV, and could allow for more people to test.

This study is designed to find out about the experiences of communities with HIV services, and whether communities could benefit from being provided with HIV self-tests.

### 2. Why are we asking you to take part in this study?

HIV self-testing has been offered in certain communities, which was determined by chance. We are interested in learning about your experiences with HIV testing, treatment and prevention. We want to understand what changes there have been in communities provided with HIV self-testing compared to communities without these services. This is important in order to learn whether HIV self-testing should be available in Malawi, and if so, how HIV self-testing should be provided.

### 3. What will happen if you decide to take part in this study?

You will be asked questions about your use of HIV services, including testing, treatment and actions that you may have taken to protect yourself from HIV. You will also be asked about your risk and perceptions of HIV, and the views of your community on HIV.

The interview will take place in your home. This will take approximately 1 hour of your time.

### 4. Who are we asking to participate?

Households in this community were selected by chance to participate in the study. We are asking all members of this household who are 15 years or older to participate, but you have been selected by chance to answer a longer set of questions.

### 5. Where do we come from?

We work at the Malawi-Liverpool-Wellcome Trust Clinical Research Programme (MLW) and Population Services International (PSI). MLW and PSI conduct research and implement projects on diseases of local importance to Malawi and the region.

### 6. What are the risks and benefits of the study?

You should feel comfortable discussing issues related to HIV and sexual health. HIV is still stigmatised in many places, and you may experience negative consequences from your family, friends or community members for participating in a study on HIV.

Your contribution will help us to understand how best to provide HIV self-testing in Malawi.

### 7. Do I have to participate in this study?

Your participation is voluntary. You may withdraw from the study at any time and without giving any reason. You can also decide to answer some questions, and not to answer other questions. If you do not agree to take part in the interview, you will not be penalised in any way.

### 8. Confidentiality

All information obtained from the study will be stored securely on paper or computer files and only researchers in this study will have access to them. We will use a number to identify you, and will only record your name on one enrollment book. The data you provide will be stored and shared, with confidentiality maintained through all data handling and storage processes.

The data you provide may be published in journals and reports so others can learn from your experience. The data may also be made available through a public data repository or to other researchers so it can be used to improve how HIV services are provided. Your personal information will not be included.

### 9. Costs

Taking part in the study will not cost you anything. If selected for the extended questionnaire, we will give you MWK 7000 to cover the cost of your time or transport.

### 10. The Ethics Committees that have approved the study are:

College of Medicine Research and Ethics Committee and London School of Hygiene and Tropical Medicine Research Ethics Committee.

### 11. What if I have any questions?

If you have any questions about HIV or about this study please feel free to ask them. If you think of any questions after we have gone please feel free to contact us by calling the following number and asking for Moses Kumwenda or Pitchaya Indravudh.

Tel: 01874628 / 01876444

Please contact the COMREC Secretariat should you wish further information about your rights, safety, and wellbeing in research:

COMREC Secretariat

College of Medicine Research and Ethics Committee

P/Bag 360, Chichiri, Blantyre 3, Malawi

Telephone: 01877 245 / 01 877 291 – ext. 334

## Consent Form

| **Statement** | **Please initial or thumbprint* each box** |
| --- | --- |
| I confirm that I have read the information sheet for the study and understand the procedures involved. I have had the opportunity to consider the information, ask questions and have these answered satisfactorily.  **OR**  I have had the information explained to me by study personnel in a language that I understand and understand the procedures involved. I have had the opportunity to consider the information, ask questions and have these answered satisfactorily. |  |
| I understand that my participation is voluntary and that I am free to withdraw at any time without giving any reason. |  |
| I understand that data collected during the study may be looked at by authorised individuals, where it is relevant to my participation in this research. I give permission for these individuals to have access to my records. |  |
| I understand that the data I provide may be shared via a public data repository or by sharing directly with other researchers, and that I will not be identifiable from this information. |  |
| I agree to take part in the study. |  |

……………………………………………… ......./......./....... ….…………..…………..………

Name of participant Date Signature or thumb print

I attest that I have explained the study information accurately, and was understood to the best of my knowledge by, the participant and that he/she has freely given their consent to participate* in the presence of the below named impartial witness (where applicable).

……………………………………………… ......./......./....... ….…………..…………..………

Name of witness Date Signature

### [*Only required if the participant is unable to read or write]

……………………………………………… ......./......./....... ….…………..…………..………

Name of interviewer Date Signature

## Assent Form

**For parent or guardian:**

| **Statement** | **Please initial or thumbprint* each box** |
| --- | --- |
| I confirm that I have read the information sheet for the study and understand the procedures involved for the young adult. I have had the opportunity to consider the information, ask questions and have these answered satisfactorily.  **OR**  I have had the information explained to me by study personnel in a language that I understand and understand the procedures involved for the young adult. I have had the opportunity to consider the information, ask questions and have these answered satisfactorily. |  |
| I understand that the participation of the young adult is voluntary and that he or she is free to withdraw at any time without giving any reason. |  |
| I understand that data collected during the study may be looked at by authorised individuals, where it is relevant to the young adult’s participation in this research. I give permission for these individuals to have access to records of the young adult. |  |
| I understand that the data the young adult provides may be shared via a public data repository or by sharing directly with other researchers, and that the young adult will not be identifiable from this information. |  |
| I agree for the young adult to take part in the study. |  |

……………………………………………… .......................

Name of young adult Age

……………………………………………… ......./......./....... ….…………..…………..………

Name of guardian Date Signature or thumb print

I attest that I have explained the study information accurately, and was understood to the best of my knowledge by, the participant and that he/she has freely given their consent to participate* in the presence of the below named impartial witness (where applicable).

……………………………………………… ......./......./....... ….…………..…………..………

Name of witness Date Signature

### [*Only required if the participant is unable to read or write]

**For young adult:**

| **Statement** | **Please initial or thumbprint* each box** |
| --- | --- |
| I confirm that I have read the information sheet for the study and understand the procedures involved. I have had the opportunity to consider the information, ask questions and have these answered satisfactorily.  **OR**  I have had the information explained to me by study personnel in a language that I understand. I have had the opportunity to consider the information, ask questions and have these answered satisfactorily. |  |
| I agree to take part in the study. |  |

……………………………………………… ......./......./....... ….…………..…………..………

Name of participant Date Signature or thumb print

I attest that I have explained the study information accurately, and was understood to the best of my knowledge by, the participant and that he/she has freely given their consent to participate* in the presence of the below named impartial witness (where applicable).

……………………………………………… ......./......./....... ….…………..…………..………

Name of witness Date Signature

### [*Only required if the participant is unable to read or write]

……………………………………………… ......./......./....... ….…………..…………..………

Name of interviewer Date Signature
